# The Vps21 signalling pathway regulates white-opaque switching and mating in *Candida albicans*

**DOI:** 10.1080/21501203.2024.2376533

**Published:** 2024-07-12

**Authors:** Fei Zhao, Han Du, Qiushi Zheng, Jian Bing, Li Tao, Clarissa J. Nobile, Guanghua Huang

**Affiliations:** a Shanghai Institute of Infectious Disease and Biosecurity, Department of Infectious Diseases, Huashan Hospital and State Key Laboratory of Genetic Engineering, School of Life Sciences, Fudan University, Shanghai, China; b Department of Molecular and Cell Biology, University of California, Merced, CA, USA; c Health Sciences Research Institute, University of California, Merced, CA, USA

**Keywords:** Vps21, Vps9, *Candida albicans*, white-opaque switching, sexual reproduction

## Abstract

*Candida albicans* is able to switch between two epigenetic cell types, namely white and opaque. Multiple conserved signalling pathways control the switch between white and opaque cell types in response to environmental changes. Here, we report the regulatory roles of the endosomal Rab family GTPase Vps21 and associated key components of the Vps21 signalling pathway in white-opaque switching and mating in *C. albicans*. Deletion of *VPS21* promoted a switch from the white to the opaque phenotype in the presence of N-acetyl-glucosamine (GlcNAc). Consistently, inactivation of the guanine nucleotide exchange factor of Vps21 (Vps9) and downstream components in the Vps21 pathway (Vps3, Vac1, and Pep12) had similar promoting effects on phenotypic switching. The mating efficiency of opaque cells is much higher than that of white cells under standard laboratory culture conditions. However, compared to the wildtype strain, the *vps21/vps21*, *vps9/vps9*, *vps3/vps3*, *vac1/vac1*, and *pep12/pep12* mutant strains exhibited dramatically reduced mating efficiencies. Quantitative RT-PCR assays demonstrated that inactivation of the Vps21 signalling pathway led to downregulation of pheromone expression and mating response pathway associated genes. Taken together, our findings indicate that the conserved Vps21 signalling pathway plays critical roles in the regulation of cell-type switching and mating in *C. albicans*.

## 1. Introduction

The opportunistic yeast pathogen *Candida albicans* can cause cutaneous diseases as well as life-threatening systemic infections in humans (Brown et al. 2012). An important characteristic of *C. albicans* is its ability to undergo morphological transitions between several phenotypes (Whiteway and Bachewich 2007; Huang 2012). This ability is associated with several biological processes including its rapid adaptation to environmental changes, virulence, and sexual mating (Soll 2009; Sudbery 2011; Huang 2012; Wang et al. 2023). For example, opaque cells are better at colonising superficial niches, whereas white cells are more virulent in systemic infection models (Soll 2009; Sudbery 2011; Huang 2012).

The bistable and heritable white-opaque switch is a typical phenotypic switching system in *C. albicans* (Slutsky et al. 1987; Soll 2009; Huang 2012). White and opaque cells exhibit significant differences in colony and cellular appearances, virulence, and mating competency (Slutsky et al. 1987; Miller and Johnson 2002; Soll 2009; Huang 2012). Under standard laboratory culture conditions, opaque cells mate one million times more efficiently than white cells (Miller and Johnson 2002). However, we recently reported that *C. albicans* white cells can mate efficiently under glucose starvation conditions (Guan et al. 2023). These findings provide new insights into the adaptive capabilities of *C. albicans* and its potential mating strategies in various environmental contexts.

Many environmental cues and signalling pathways are involved in the regulation of white-opaque switching and mating in *C. albicans* (Slutsky et al. 1987; Soll 2009; Huang 2012). For example, high levels of CO_2_, acidic pH, and N-acetyl-glucosamine (GlcNAc) promote white-to-opaque switching, while high temperatures and basic pH favour the white cell development (Huang et al. 2009, 2010; Lohse and Johnson 2009; Soll 2009; Huang 2012; Sun et al. 2015). Multiple signalling pathways are involved in the regulation of this environmental cues-induced phenotypic switch. For example, the cAMP-dependent PKA signalling pathway, the Ste11-Hst7-Cek1/Cek2-mediated MAPK cascade pathway, and the osmotic sensing pathway play critical roles in the regulation of white-opaque switching and mating in *C. albicans* (Huang et al. 2010; Ramirez-Zavala et al. 2013; Liang et al. 2014). In activation of the cAMP-dependent PKA signalling pathway reduced the efficiency of white-to-opaque switching, while overexpression of the key components of the Ste11-Hst7-Cek1/Cek2-mediated MAPK pathway promotes the development of opaque cells (Huang et al. 2010; Ramirez-Zavala et al. 2013; Liang et al. 2014).

The endosomal GTPase Vps21-mediated signalling pathway is conserved in fungi and involved in the regulation of a number of important biological processes including vacuolar biogenesis and trafficking, autophagy, and morphological transitions (Rieder et al. 1996; Gerrard et al. 2000; Palmer et al. 2003; Chen et al. 2014). Vps9 is a guanine nucleotide exchange factor (GEF) for Vps21 and facilitates the exchange of GTP for GDP on the guanine nucleotide binding site, leading to GTPase activation (Johnston et al. 2013; Chen et al. 2014; Borchers et al. 2021). The CORVET tethering complex components Vps3, Vac1, and Pep12 (the soluble N-ethylmaleimide-sensitive factor attachment protein receptor, t-SNARE) are major downstream factors in the Vps21 signalling pathway (Johnston et al. 2013; Chen et al. 2014; Borchers et al. 2021). Large vacuoles are commonly observed in *C. albicans* opaque cells and shmoos, the elongated morphology yeast cells adopt during mating (Anderson et al. 1989; Liang et al. 2020). In this study, we investigated whether the Vps21 signalling pathway plays a role in the regulation of white-opaque switching and mating in *C. albicans*. Our findings show that inactivation of the Vps21 signalling pathway promotes the white-to-opaque switch but represses mating in *C. albicans*. Furthermore, disruption of the Vps21 signalling pathway leads to downregulation of mating-associated gene expression, which could directly affect the mating response in *C. albicans*.

## 2. Materials and methods

### 2.1. Strains and culture conditions

YPD medium (1% yeast extract, 2% peptone, 2% glucose; 2% agar added for solid medium, w/v) was used for regular growth of *C. albicans* cells. Modified Lee’s glucose (pH 6.8) and Lee’s GlcNAc (pH 6.8) media containing phloxine B (5 μg/mL) were used for white-opaque switching and quantitative mating assays (Huang et al. 2010; Li et al. 2023). Synthetic complete [SC, 0.67% yeast nitrogen base with ammonium sulphate and without amino acids (YNB), 2% glucose, and corresponding amino acids] medium was used for selective growth in quantitative mating assays. The strains used in this study are listed in supplementary Table S1.

### 2.2. Plasmid and strain construction

To delete *VPS21*, *VPS9*, *VPS3*, *VAC1*, or *PEP12* in *C. albicans*, the fusion PCR recombination strategy was used as described by the Noble group (Noble and Johnson 2005). To delete the first copy of each gene, a long fusion PCR fragment of *CdHIS1* flanked by ~400 bp upstream and downstream sequences of the target gene was transformed into strain SN152 [*MTL***a**/α, a derivative of SC5314 (Noble and Johnson 2005)], generating a heterozygous mutant. A fusion PCR fragment of *CmLEU2* or *CaARG4* flanked by ~400 bp upstream and downstream sequences of the target gene was then transformed into a heterozygous mutant, generating a homozygous mutant. For white-opaque switching and mating assays, the deletion mutants of *VPS21*, *VPS9*, *VPS3*, *VAC1*, and *PEP12* were converted to *MTL***a**/Δ or *MTL*Δ/α strains by deleting one allele of the *MTL* locus. The linearising plasmid L23.14 [a pSFS2A-based *MTL* locus deletion plasmid (Park and Morschhauser 2005; Xie et al. 2013)] was used for deletion of the *MTL* locus.

The method for construction of the reconstituted strains was used as described previously (Li et al. 2023). To construct the *VPS21* complementation plasmid, a fragment of *VPS21*-3’-UTR (untranslated region) was amplified from genomic DNA of strain SC5314, digested with two restriction enzymes, *Xho*I (*Sal*I isocaudamer) and *Bgl*II, and subcloned into the *Sal*I/*BgI*II sites of plasmid pNIM1, generating pNIM1-*VPS21*-3’-UTR. Then, a fragment containing *VPS21*-5’-UTR and its ORF was amplified from genomic DNA of strain SC5314, digested with two restriction enzymes, *Sal*I and *BamH*I (*BgI*II isocaudamer), and subcloned into the *Sal*I/*BgI*II sites of plasmid pNIM1-*VPS21*-3’-UTR, generating the complementation plasmid *VPS21*p-*VPS21*. To construct the *VPS21*^*Q69L*^ or *VPS21*^*S24N*^ complementation plasmid, a fragment containing *VPS21*^Q69L^ or *VPS21*^S24N^-5’-UTR and ORF was obtained by site-directed mutagenesis (Johnston et al. 2013) and subcloned into the *Sal*I/*BamH*I sites of plasmid pNIM1-*VPS21*-3’-UTR, generating the complementation plasmid *VPS21*p-*VPS21*^Q69L^ or *VPS21*p-*VPS21*^S24N^. Similarly, we constructed the complementation plasmid for *VPS3* called *VPS3*p-*VPS3* using the same strategy. To construct the *VPS9* complementation plasmid, a fragment of *VPS9*-3’-UTR was amplified and subcloned into the *EcoR*V/*Hind*III sites of the plasmid BES116 (Feng et al. 1999). The *caSAT1* gene (nourseothricin resistance marker adapted for *C*. *albicans*) was amplified from the plasmid pSFS2A (Reuss et al. 2004) and inserted into the *Kpn*I site of the plasmid BES116-VPS9-3’-UTR. Then, the fragment containing the *VPS9*-5’-UTR and ORF was amplified from *C. albicans* genomic DNA and subcloned into the *Hind*III/*Kpn*I sites of the plasmid BES116-*VPS9*-3’-UTR-SAT1, generating the complementation plasmid *VPS9*p-*VPS9*. To construct the *VAC1* complementation plasmid, a fragment of *VAC1*-3’-UTR was amplified and subcloned into the *Kpn*I/*EcoR*V site of the plasmid BES116. The fragment containing *VAC1*-5’-UTR and its ORF was amplified from *C. albicans* genomic DNA and subcloned into the *EcoR*V/*Pst*I site of the plasmid BES116-*VAC1*-3’-UTR. Then, the selectable marker *caSAT1* was inserted into the *Pst*I site of the plasmid BES116-*VAC1*-5’-UTR-ORF-3’-UTR, generating the complementation plasmid *VAC1*p-*VAC1*. To construct the *PEP12* complementation plasmid, a fragment of *PEP12*-3’-UTR was amplified and subcloned into the *Kpn*I/*Hind*III site of the plasmid BES116. The fragment containing *PEP12-*5’-UTR and its ORF were amplified from genomic DNA and subcloned into the *Hind*III/*Pst*I sites of the plasmid BES116-*PEP12*-3’-UTR. Then, the selectable marker *caSAT1* was inserted into the *Pst*I site of the plasmid BES116-*PEP12*-5’-UTR-ORF-3’-UTR, generating the complementation plasmid *PEP12*p-*PEP12*. Finally, the constructed complementation plasmids were linearised with restriction enzymes (*Sal*I for pNIM1-based plasmids, *Hind*III for the plasmid *VPS9p-VPS9* and *PEP12*p-*PEP12*, and *EcoR*V for plasmid *VAC1p-VAC1*) and transformed into the corresponding mutant strains, generating the reconstituted complemented strains (*vps21*/*vps21 + VPS21*, *vps21*/*vps21 + VPS21*^*Q69L*^, *vps21*/*vps21 + VPS21*^*S24N*^, *vps9*/*vps9 + VPS9*, *vps3*/*vps3 + VPS3*, *vac1*/*vac1 + VAC1*, and *pep12*/*pep12 + PEP12*). The primer sequences used for PCR assays are presented in Table S2.

### 2.3. White-opaque switching assay

White-opaque switching assays were performed as described previously (Huang et al. 2010). *C. albicans* opaque or white cells were initially plated onto Lee’s glucose medium and incubated at 25 °C for 7 days. For quantitative switching assays, homogeneous white or opaque colonies were then selected and replated onto Lee’s glucose or Lee’s GlcNAc medium (pH 6.8) and cultured at 25 °C for 5 days. Three biological repeats were performed. Switching frequency = average percentage of colonies with the alternative phenotype or sectored colonies ± standard deviation (SD).

### 2.4. Quantitative mating assay

Quantitative mating assays were performed as previously described (Miller and Johnson 2002; Xie et al. 2013; Li et al. 2023). Opaque cells were first grown on Lee’s glucose medium at 25 °C for 5 days. Approximately 2 × 10^7^ cells of *MTL***a**/Δ and *MTL*Δ/α strains were mixed, spotted onto Lee’s glucose and Lee’s GlcNAc medium (pH 6.8) and incubated at 25 °C for 3 days. The mating mixtures were then resuspended, diluted, and plated onto SC media (without arginine, leucine, or both).

### 2.5. Quantitative real-time RT-PCR assays

*Candida albicans* cells (*MTL***a**/Δ or *MTL*Δ/α) were initially cultured on Lee’s glucose medium at 25 °C for 5 days. For pheromone treatment assays, approximately 2 × 10^7^ opaque cells (*MTL***a**/Δ) were inoculated into liquid Lee’s glucose medium (4 mL) containing 50 μmol/L α-pheromone and incubated at 25 °C for 12 h to examine the relative expression levels of pheromone response associated genes. To examine the relative expression levels of mating associated genes in *MTL*Δ/α cells, approximately 2 × 10^7^ opaque cells (*MTL*Δ/α) were incubated in 4 mL of liquid Lee’s glucose medium (pH 6.8) and cultured at 25 °C for 24 h. Fungal cells (*MTL***a**/Δ or *MTL*Δ/α) were harvested, and total RNA was extracted using RNA purification kit (Thermo Scientific, K0732, Thermo Scientific, Waltham, USA). Total RNA (1 μg per sample) was used to synthesise cDNA with RevertAid H Minus Reverse Transcriptase (Thermo Fisher Scientific Inc., Shanghai, China). The expression level of the *C. albicans ACT1* gene was used for normalisation.

## 3. Results

### 3.1. *Vps21 regulates white-opaque switching in* C. albicans

**Figure 1.**
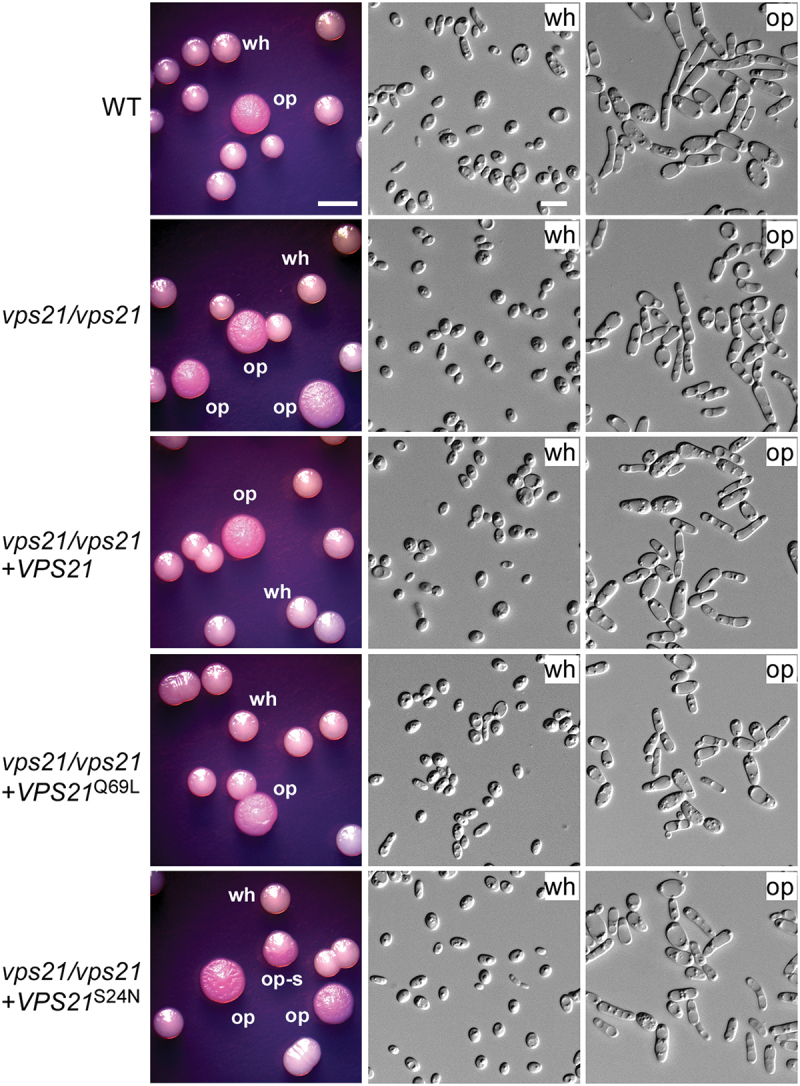
Function of Vps21 and its variants in the regulation of white-to-opaque switching. White cells of the WT control, *vps21*/*vps21* mutant, and reconstituted strains were initially grown on Lee’s glucose medium (pH 6.8) at 25 °C for 7 days. *Candida albicans* cells of homogeneous white colonies were replated onto Lee’s GlcNAc medium (pH 6.8) and incubated at 25 °C for 5 days. wh, white; op, opaque; op-s, opaque-sectored (colonies containing both white and opaque cells). Scale bar for cells, 10 μm; Scale bar for colonies, 2 mm. Strains used: WT (FDZF208); *vps21*/*vps21* (FDZF263); *vps21*/*vps21 + VPS21* (FDZF489); *vps21*/*vps21 + VPS21*^S24N^ (FDZF475); and *vps21*/*vps21 + VPS21*^Q69L^ (FDZF473). The mating type of all strains used was *MTL***a**/Δ. The switching frequencies are presented in Table 1.

Opaque cells of *C. albicans* often contain a large vacuole (Anderson and Soll 1987; Soll 2009). Since the Vps21 signalling pathway is associated with vacuolar trafficking and protein sorting in fungi (Stack et al. 1995; Gerrard et al. 2000; Toshima et al. 2014), we set out to investigate whether this pathway plays a role in the regulation of *C. albicans* white-opaque switching. We generated a *vps21/vps21* deletion mutant strain in *C. albicans* using the fusion PCR homologous recombination strategy (Noble and Johnson 2005). White-opaque switching assays demonstrated that compared to the WT strain [(14.6 ± 0.2)%] and reconstituted strain (*vps21/vps21* + *VPS21*) [(10.9 ± 1.0)%], the *vps21/vps21* mutant strain [(30.9 ± 2.3)%] exhibited a significantly higher white-to-opaque switching frequency on Lee’s GlcNAc medium (Figure 1 and Table 1). However, both the WT and *vps21/vps21* mutant strains demonstrated low frequencies of white-to-opaque switching on Lee’s glucose medium (Table 1). The opaque-to-white switching frequencies of the WT strain, *vps21/vps21* mutant strain, and reconstituted strain (*vps21/vps21* + *VPS21*) were comparable on both Lee’s glucose and Lee’s GlcNAc media (Table 1).

**Table 1.** White-opaque switching frequencies of the WT control and mutant strains of the Vps21 signalling pathway.

Strain	Wh-to-op (%)		Op-to-wh (%)	
	Lee’s GlcNAc	Lee’s glucose	Lee’s GlcNAc	Lee’s glucose
WT	14.6 ± 0.2	<0.2	0.7 ± 0.8	7.3 ± 3.4
*vps21*/*vps21*	30.9 ± 2.3	<0.4	<0.3	8.3 ± 3.7
*vps21*/*vps21* *+* *VPS21*	10.9 ± 1.0	<0.2	<0.3	5.4 ± 2.5
*vps21/vps21 + VPS21*^Q69L^	12.4 ± 0.6	<0.2	<0.2	8.9 ± 0.6
*vps21/vps21 + VPS21*^S24N^	30.8 ± 3.0	<0.2	<0.2	7.6 ± 1.2
*vps9*/*vps9*	44.1 ± 3.5	<0.2	<0.3	19.8 ± 3.8
*vps9*/*vps9* *+* *VPS9*	19.0 ± 1.2	<0.3	0.3 ± 0.6	7.8 ± 2.6
*vps3*/*vps3*	94.3 ± 3.1	<0.3	<0.2	27.6 ± 2.1
*vps3*/*vps3 + VPS3*	8.1 ± 0.6	<0.3	<0.2	6.5 ± 2.1
*vac1*/*vac1*	91.7 ± 3.4	<0.3	0.3 ± 0.6	31.4 ± 4.4
*vac1*/*vac1* *+* *VAC1*	8.9 ± 4.2	<0.4	1.5 ± 1.2	9.8 ± 2.5
*pep12*/*pep12*	96.9 ± 1.8	<0.2	<0.3	32.4 ± 4.5
*pep12*/*pep12* *+* *PEP12*	8.1 ± 1.7	<0.3	<0.2	15.4 ± 5.3

White and opaque cells of the WT control and mutant strains were initially grown on Lee’s glucose medium (pH 6.8) for 7 days. *Candida albicans* cells of homogeneous white or opaque colonies were replated onto Lee’s glucose (pH 6.8) or Lee’s GlcNAc medium (pH 6.8) and incubated at 25 °C for 5 days. Wh-to-op (%), white-to-opaque switching frequency (percentage); op-to-wh (%), opaque-to-white switching frequency (percentage); <, no colonies with the alternative phenotype were observed. Strains used: WT (FDZF208); *vps21*/*vps21* (FDZF263); *vps9*/*vps9* (FDZF213); *vps3*/*vps3* (FDZF259); *vac1*/*vac1* (FDZF531); *pep12*/*pep12* (FDZF546); *vps21*/*vps21 + VPS21* (FDZF489); *vps21*/*vps21 + VPS21*^S24N^ (FDZF475); *vps21*/*vps21 + VPS21*^Q69L^ (FDZF473); *vps9*/*vps9 + VPS9* (FDZF491); *vps3*/*vps3 + VPS3* (FDZF493); *vac1*/*vac1 + VAC1* (FDZF548); and *pep12*/*pep12 + PEP12* (FDZF549). The mating type of all strains used was *MTL***a**/Δ.

**Figure 2.**
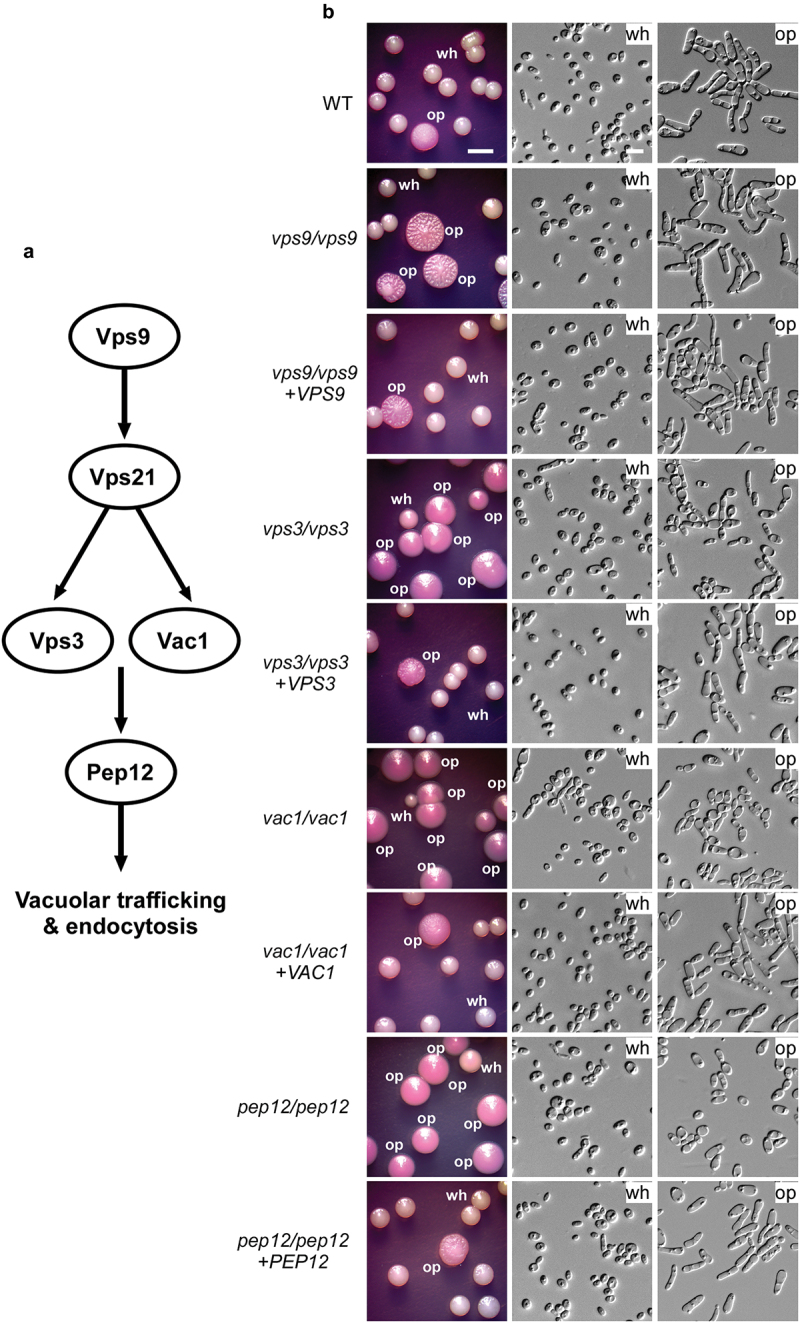
Deletion of *VPS9*, *VPS3*, *VAC1*, and *PEP12* promotes white-to-opaque switching. (a) Schematic diagram of the Vps21 signaling pathway. Vps9, a guanine nucleotide exchange factor (GEF) of Vps21; Vps3, a CORVET tethering complex component; Vac1, a vesicle transport protein; and Pep12, a t-SNARE involved in prevacuolar trafficking. (b) White-to-opaque switching assays. White cells of the WT control, *vps9*/*vps9*, *vps3*/*vps3*, *vac1*/*vac1*, and *pep12*/*pep12* mutant, and corresponding reconstituted strains were initially grown on Lee’s glucose medium (pH 6.8) for 7 days. *Candida albicans* cells of homogeneous white colonies were replated onto Lee’s GlcNAc medium (pH 6.8) and incubated at 25 °C for 5 days. Wh, white; op, opaque. Scale bar for cells, 10 μm; Scale bar for colonies, 2 mm. Strains used: WT (FDZF208); *vps9*/*vps9* (FDZF213); *vps3*/*vps3* (FDZF259); *vac1*/*vac1* (FDZF531); *pep12*/*pep12* (FDZF546); *vps9*/*vps9 + VPS9* (FDZF491); *vps3*/*vps3 + VPS3* (FDZF493); *vac1*/*vac1 + VAC1* (FDZF548); and *pep12*/*pep12 + PEP12* (FDZF549). The mating type of all strains used was *MTL***a**/Δ. The switching frequencies are presented in Table 1.

GEF activator, which facilitates the switch between an inactive GDP-bound state and an active GTP-bound state (Chen et al. 2014). We next ectopically expressed putative GTP-locked (active, *VPS21*^Q69L^) and GDP-locked (inactive, *VPS21*^S24N^) states in the *vps21/vps21* mutant strain and examined the white-to-opaque switching frequencies. As expected, the *VPS21*^Q69L^ active strain [(12.4 ± 0.6)%] exhibited a comparable switching frequency to that of the WT control strain [(14.6 ± 0.2)%], whereas the *VPS21*^S24N^ inactive strain [(30.8 ± 3.0)%] showed an increased frequency on Lee’s GlcNAc medium compared to the WT control strain (Figure 1 and Table 1). Consistently, we observed that deletion of the GEF activator-encoding gene *VPS9* had a similar promoting effect on the induction of the opaque phenotype in *C. albicans* under the same culture condition (Figure 2 and Table 1). Taken together, these results suggest that the Vps21 GTPase plays a critical role in regulating white-opaque switching in *C. albicans*.

### 3.2. Vps3, Vac1, and Pep12 regulate white-opaque switching

Vps3, Vac1, and Pep12 are downstream factors in the Vps21 signalling pathway (Chen et al. 2014). To investigate their roles in white-opaque switching, we generated *vps3*/*vps3*, *vac1*/*vac1*, and *pep12*/*pep12* mutant strains in *C. albicans* and performed white-opaque switching assays. As shown in Figure 2 and Table 1, all three mutant strains exhibited extremely high white-to-opaque switching frequencies on Lee’s GlcNAc medium [(94.3 ± 3.1)% for the *vps3*/*vps3* mutant strain, (91.7 ± 3.4)% for the *vac1*/*vac1* mutant strain, and (96.9 ± 1.8)% for the *pep12*/*pep12* mutant strain)]. These mutant strains also had relatively high opaque-to-white switching frequencies on Lee’s glucose medium (approximately 30%, Table 1), suggesting that deletion of these genes has a bidirectional effect on promoting phenotypic transitions. Similar to the *vps21*/*vps21* and *vps9/vps9* mutant strains, the white phenotypes of the *vps3*/*vps3*, *vac1*/*vac1*, and *pep12*/*pep12* mutant strains were stable on Lee’s glucose medium (switching frequency < 0.4%), while the opaque phenotypes of these mutant strains were stable on Lee’s GlcNAc medium (switching frequency < 0.4%). As expected, the switching frequencies between white and opaque phenotypes of the *VPS3-*, *VAC1-*, and *PEP12*-reconstituted strains were comparable to those of the WT control strain. Interestingly, we found that the *vps3/vps3*, *vac1/vac1*, and *pep12/pep12* mutants exhibited a much stronger white-to-opaque switching ability than the *vps21/vps21* mutant, indicating that some other signalling pathways or regulators are involved in this regulation. There could be cross-talks and inter-regulations among these pathways in the regulation of phenotypic switching. Taken together, our results suggest that the Vps21 downstream factors Vps3, Vac1, and Pep12 also play roles in the regulation of white-opaque switching in *C. albicans*.

### 3.3. *The Vps21 signaling pathway regulates sexual mating in* C. albicans

Since white-opaque switching regulates mating in *C. albicans* and closely related species (Pujol et al. 2004; Porman et al. 2011; Xie et al. 2013), we next tested whether the Vps21 signalling pathway plays a role in the regulation of mating. As shown in Table 2, quantitative mating assays demonstrated that the inactivation of Vps21 or Vps9 led to an approximately 8- to 193-fold decrease in mating efficiency on Lee’s glucose or Lee’s GlcNAc medium (compared to the WT**a** × WTα control cross). Notably, the *vps3/vps3*, *vac1/vac1*, and *pep12/pep12* mutant strains exhibited dramatically decreased mating efficiencies on both media (decreased by three to five orders of magnitude). The mating efficiency of these mutants was particularly low on Lee’s GlcNAc medium (Table 2). These results imply that the Vps21 signalling pathway is required for efficient mating in *C. albicans*.

**Table 2.** Mating efficiencies of the WT control and mutant strains of the Vps21 signalling pathway.

Cross (**a** x α)	Mating efficiency	
	Lee’s glucose	Lee’s GlcNAc
WT **a** x WT α	(4.7 ± 1.9) × 10^−2^	(5.6 ± 1.9) × 10^−3^
*vps21*/*vps21***a** x *vps21*/*vps21* α	(5.7 ± 0.6) × 10^−3^	(2.9 ± 0.6) × 10^−5^
*vps9*/*vps9***a** x *vps9*/*vps9* α	(4.2 ± 3.0) × 10^−3^	(5.1 ± 1.6) × 10^−5^
*vps3*/*vps3***a** x *vps3*/*vps3* α	(9.0 ± 0.8) × 10^−5^	(2.2 ± 3.7) × 10^−8^
*vac1*/*vac1***a** x *vac1*/*vac1* α	(8.2 ± 3.1) × 10^−5^	< (4.2 ± 1.6) × 10^−8^
*pep12*/*pep12***a** x *pep12*/*pep12* α	(7.8 ± 3.0) × 10^−5^	(8.0 ± 2.9) × 10^−8^

Opaque *MTL***a**/Δ and *MTL*Δ/α cells were mixed and incubated on Lee’s glucose (pH 6.8) or Lee’s GlcNAc (pH 6.8) medium at 25 °C for 3 days. The mixtures were then plated onto selective media for mating efficiency assays. <, no colonies with the alternative phenotype were observed. Strains used: WT**a** (FDZF208); WTα, (FDZF171); *vps21/vps21***a** (FDZF263); *vps21/vps21*α (FDZF266); *vps9*/*vps9***a** (FDZF213); *vps9*/*vps9*α (FDZF212); *vps3*/*vps3***a** (FDZF259); *vps3*/*vps3*α (FDZF250); *vac1*/*vac1***a** (FDZF531); *vac1*/*vac1*α (FDZF533); *pep12*/*pep12***a** (FDZF546); and *pep12*/*pep12*α (FDZF534).

### 3.4. Deletion of genes associated with the Vps21 signaling pathway downregulates the expression of mating-related genes

**Figure 3.**
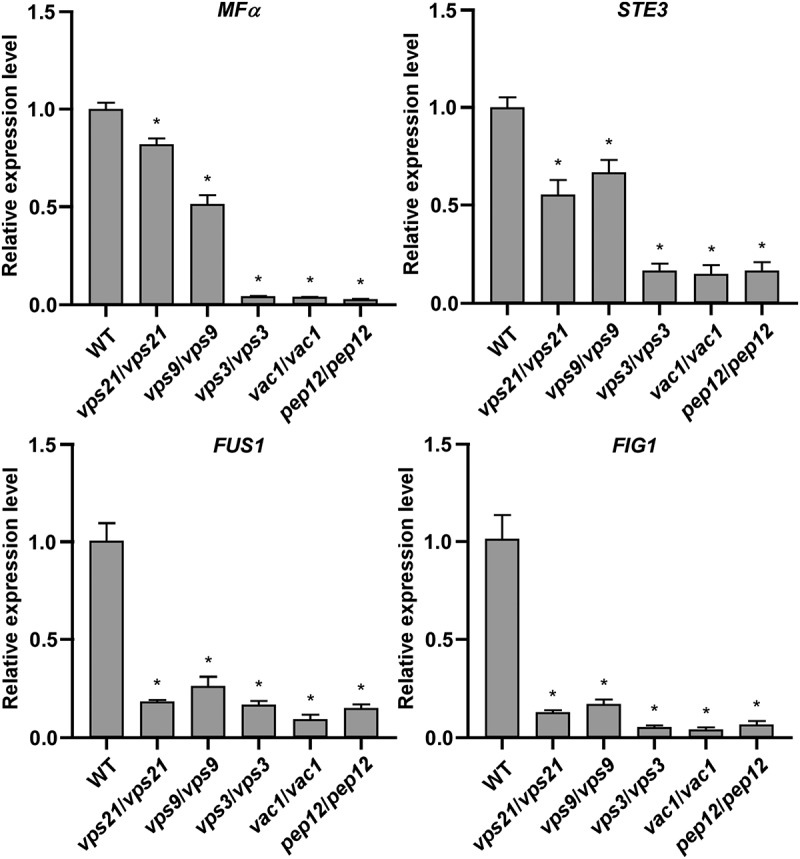
Relative transcriptional expression of mating associated genes in the WT control and mutant strains of the Vps21 signalling pathway. Opaque cells of the WT control, *vps21/vps21*, *vps9*/*vps9*, *vps3*/*vps3*, *vac1*/*vac1*, and *pep12*/*pep12* mutant strains were incubated in liquid Lee’s glucose medium (pH 6.8) for 24 hours at 25 °C. Total RNA was extracted for quantitative RT-PCR assays. The expression level of *ACT1* was used for normalisation. The average value of the WT control strain for each gene was set as “1”. “*” indicates significant difference between the WT control strain and mutant strain (*p* < 0.05, two-tailed Student’s *t*-test). Strains used: WT (FDZF171); *vps21*/*vps21* (FDZF266); *vps9*/*vps9* (FDZF212); *vps3*/*vps3* (FDZF250); *vac1*/*vac1* (FDZF533); and *pep12*/*pep12* (FDZF534). The mating type of all strains used was *MTL*Δ/α.

To explore the potential mechanism of the Vps21 signalling pathway in regulating mating in *C. albicans*, we performed quantitative RT-PCR assays to test the relative expression levels of mating-associated genes. As shown in Figures 3 and S1, the relative expression levels of *MFα* (encoding the α-pheromone precursor) (Bennett et al. 2003; Lockhart et al. 2003; Panwar et al. 2003), *STE3* (encoding the **a**-pheromone receptor) (Chen et al. 2002; Magee et al. 2002), *FUS1* (encoding a membrane protein required for cell fusion), *FIG1* (encoding a membrane protein required for efficient mating and cell fusion), *CPH1* (a *Saccharomyces cerevisiae STE12* homolog required for mating) (Liu et al. 1994), and the mating-specific MAPK pathway-associated genes (including *CST20, STE11*, *HST7*, *CEK1*, and *CEK2*) (Chen et al. 2002; Magee et al. 2002) were significantly downregulated in the *vps21/vps21*, *vps9/vps9*, *vps3/vps3*, *vac1/vac1*, and *pep12/pep12* mutant strains (compared to the WT control strain). The changes in relative expression levels of these mating-associated genes largely corresponded to the decreased mating efficiencies in the Vps21 signalling pathway mutant strains.

**Figure 4.**
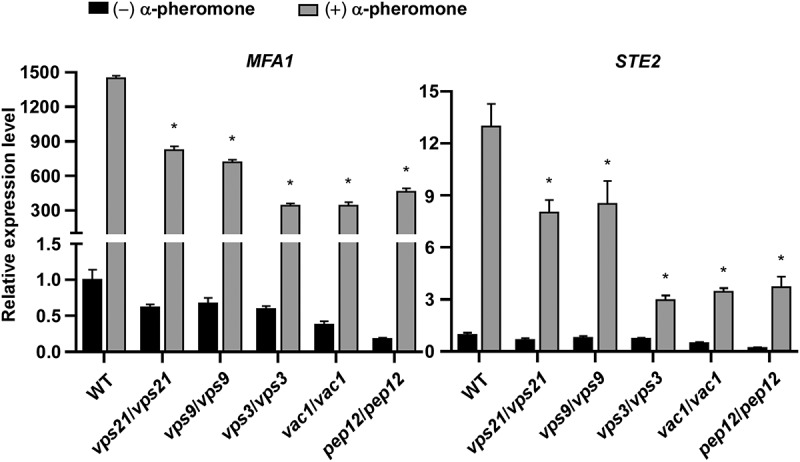
Relative transcriptional expression of *MFA1* and *STE2* in the absence and presence of α-pheromone in the WT control and mutant strains of the Vps21 signalling pathway. Opaque cells of the WT control, *vps21/vps21*, *vps9*/*vps9*, *vps3*/*vps3*, *vac1*/*vac1*, and *pep12*/*pep12* mutant strains were incubated in liquid Lee’s glucose medium (pH 6.8) and treated with or without synthetic α-pheromone (50 μg/mL) for 12 hours at 25 °C. Total RNA was extracted for quantitative RT-PCR assays. The expression level of *ACT1* was used for normalisation. The average value of the WT control strain for each gene without pheromone treatment was set as “1”. “*” indicates significant difference between the WT control strain and mutant strain (*p* < 0.05, two-tailed Student’s *t*-test). Strains used: WT (FDZF208); *vps21*/*vps21* (FDZF263); *vps9*/*vps9* (FDZF213); *vps3*/*vps3* (FDZF259); *vac1*/*vac1* (FDZF531); and *pep12*/*pep12* (FDZF546). The mating type of all strains used was *MTL***a**/Δ.

We next examined the induction of mating-associated genes [*MFA1* (Dignard et al. 2007) and *STE2* (Chen et al. 2002; Magee et al. 2002)] in response to synthetic α-pheromone exposure. Consistently, we observed that inactivation of the Vps21 signalling pathway remarkably suppressed the expression of *MFA1* and *STE2* in the presence of α-pheromone (Figure 4). In summary, these findings indicate that the Vps21 signalling pathway is critical for activation of the mating response pathway and is required for efficient mating in *C. albicans*.

## 4. Discussion

To mate efficiently under standard laboratory culture conditions, *C. albicans* must first undergo a morphological switch from white to opaque cell type (Miller and Johnson 2002). Many environmental factors and genes are involved in the regulation of white-opaque switching and mating in *C. albicans* (Lohse and Johnson 2009; Soll 2009; Huang 2012; Guan et al. 2019; Li et al. 2023). In this study, we report that the conserved Vps21 signalling pathway regulates both white-opaque switching and sexual mating in *C. albicans*. Inactivation of the major components of the Vps21 signalling pathway (including Vps21, Vps9, Vps3, Vac1, and Pep12) promoted white-to-opaque switching on Lee’s GlcNAc medium but repressed mating in *C. albicans* (Figures 1 and 2, Tables 1 and 2). There is an obvious negative correlation between white-to-opaque switching frequencies and mating efficiencies in the *vps21/vps21*, *vps9/vps9*, *vps3/vps3*, *vac1/vac1*, and *pep12/pep12* mutant strains. For example, the *vps3/vps3*, *vac1/vac1*, and *pep12/pep12* mutant strains exhibited high white-to-opaque switching frequencies (>90%) but mated poorly (Tables 1 and 2). It remains to be investigated as to why the Vps21 signalling pathway plays opposing roles in the regulation of these two canonically linked biological processes.

A major function of the Vps21 signalling pathway in fungi is in vacuolar biogenesis and trafficking (Stack et al. 1995; Rieder et al. 1996; Palmer et al. 2003; Toshima et al. 2014). Previous studies have demonstrated that *C. albicans* opaque cells and projected opaque cells (“shmoos” formed during mating) often contain large vacuoles (Anderson and Soll 1987; Liang et al. 2020). Given the global regulatory roles of the Vps21 signalling pathway and vacuoles, it is reasonable that impairment of this pathway or the vacuole itself could affect white-opaque switching and mating in *C. albicans*. Consistent with this hypothesis, a previous study found that Vps21-mediated vacuolar trafficking controls cellular morphogenesis and is important for hyphal development (Johnston et al. 2013). However, the mechanistic association between the Vps21 signalling pathway, vacuoles, phenotypic switching, and mating remains to be established in future studies.

We found that inactivation of genes related to the Vps21 signalling pathway had similar effects on the repression of mating in *C. albicans* on Lee’s glucose and Lee’s GlcNAc medium (Table 2). We observed that the mutant strains of this pathway exhibited distinct white-opaque switching frequencies under different culture conditions (Table 1). On Lee’s glucose medium, deletion of these genes (especially *VPS3*, *VAC1*, and *PEP12*) had promoting effects on the induction of the white cell type. However, on Lee’s GlcNAc medium, inactivation of the Vps21 signalling pathway induced the opaque phenotype. These results suggest that the regulatory roles of this pathway in white-opaque switching could be different under different culture conditions. One possible explanation for these observed differences is that GlcNAc is an opaque inducer via the activation of the cAMP/PKA signalling pathway which could have an additive effect on the induction of the opaque phenotype (Huang et al. 2010). Quantitative RT-PCR assays demonstrated that deletion of *VPS21*, *VPS9*, and their downstream factors led to reduced expression levels of mating-associated genes in *C. albicans* (Figure 3). This reduced transcriptional expression could directly repress the mating response (Figure 4).

In summary, our study indicates that the Vps21 signalling pathway plays a critical role in the control of both white-opaque transitions and sexual mating in *Candida albicans*. Additionally, the ability of mutant strains of the Vps21 signalling pathway to induce the mating-competent opaque phenotype depends on specific culture conditions.‬‬ Deletions of the genes associated with this pathway lead to a repressing effect on sexual mating, perhaps through downregulation of expression of mating-associated genes. Our findings shed new light on the biological roles of the Vps21 signalling pathway in the regulation of morphological transitions and sexual mating in *C. albicans.*

## Supplementary Material

Supplemental Material
