# Supplementary material for: The Vps21 signalling pathway regulates white-opaque switching and mating in Candida albicans
Source: Mycology. 2024 Jul 12;16(1):357–68. doi: 10.1080/21501203.2024.2376533 (PMC11899209; doi:10.1080/21501203.2024.2376533)
Supplement: Supplemental Material [file TMYC_A_2376533_SM4730.zip › revised_Table_S1_Strains_used_0411_2024.docx]

**Table S1. Strains used in this study**

| **Strain name** | **Strain** | **Parent strain** | **Genotype** | **Reference** |
| --- | --- | --- | --- | --- |
| SN152 | SN152**a/**α  (*his1/his1*  *arg4*/*arg4*  *leu2*/*leu2*) | CAI4 | *MTL****a***/*MTL**α*  *ura3::imm434::URA3*/*ura3::imm434 iro::*  *IRO1*/*iro1::imm434 his1::hisG*/*his1::hisG leu2::hisG*/*leu2::hisG arg4::hisG*/*arg4::hisG* | (Noble et al. 2010) |
| SN152**a** | SN152**a**  (*his1/his1*  *arg4*/*arg4*  *leu2*/*leu2*) | SN152 | *MTL****a***/*mtlα::SAT1*  *ura3::imm434::URA3*/*ura3::imm434 iro::*  *IRO1*/*iro1::imm434* *his1::hisG*/*his1::hisG leu2::hisG*/*leu2::*  *hisG arg4::hisG*/*arg4::hisG* | (Tao et al. 2017) |
| SN152α | SN152α  (*his1/his1*  *arg4*/*arg4*  *leu2*/*leu2*) | SN152 | *mtla::SAT1*/*MTLα*  *ura3::imm434::URA3*/*ura3::imm434 iro::IRO1*/*iro1::imm434 his1::hisG*/*his1::hisG leu2::hisG*/*leu2::hisG arg4::hisG*/*arg4::hisG* | This study |
| FDZF208 | SN152**a** (*leu2*/*leu2*) | SN152**a** | *MTL****a***/*mtlα::SAT1*  *ura3::imm434::URA3*/*ura3::imm434 iro::IRO1*/*iro1::imm434 his1::hisG*/*his1::hisG::CdHIS1*  *leu2::hisG*/*leu2::hisG*  *arg4::hisG*/*arg4::hisG::ARG4* | This study |
| FDZF171 | SN152α  (*arg4*/*arg4*) | SN152α | *mtla::SAT1*/*MTLα*  *ura3::imm434::URA3*/*ura3::imm434 iro::*  *IRO1*/*iro1::imm434*  *his1::hisG*/*his1::hisG::CdHIS1*  *leu2::hisG*/*leu2::hisG::CmLEU2*  *arg4::hisG*/*arg4::hisG* | This study |
| FDZF104 | *vps21*/*vps21* **a/**α (*leu2*/*leu2*) | SN152 | As SN152, but *vps21::HIS1*/*vps21::*  *ARG4 MTL***a**/α | This study |
| FDZF109 | *vps21*/*vps21* **a/**α (*arg4*/*arg4*) | SN152 | As SN152, but *vps21::HIS1*/*vps21::*  *LEU2 MTL***a**/α | This study |
| FDZF263 | *vps21*/*vps21* **a** (*leu2*/*leu2*) | FDZF104 | As SN152, but *vps21::HIS1*/*vps21::*  *ARG4 MTL***a**/Δ | This study |
| FDZF266 | *vps21*/*vps21* α (*arg4*/*arg4*) | FDZF109 | As SN152, but *vps21::HIS1*/*vps21::*  *LEU2 MTL*Δ/α | This study |
| FDZF165 | *vps9*/*vps9* **a/**α (*leu2*/*leu2*) | SN152 | As SN152, but *vps9::HIS1*/*vps9::*  *ARG4 MTL***a**/α | This study |
| FDZF151 | *vps9*/*vps9* **a/**α (*arg4*/*arg4*) | SN152 | As SN152, but *vps9::HIS1*/*vps9::*  *LEU2 MTL***a**/α | This study |
| FDZF213 | *vps9*/*vps9* **a** (*leu2*/*leu2*) | FDZF165 | As SN152, but *vps9::HIS1*/*vps9::*  *ARG4 MTL***a**/Δ | This study |
| FDZF212 | *vps9*/*vps9* α (*arg4*/*arg4*) | FDZF151 | As SN152, but *vps9::HIS1*/*vps9::*  *LEU2 MTL*Δ/α | This study |
| FDZF162 | *vps3*/*vps3* **a/**α (*leu2*/*leu2*) | SN152 | As SN152, but *vps3::HIS1*/*vps3::*  *ARG4 MTL***a**/α | This study |
| FDZF158 | *vps3*/*vps3* **a/**α (*arg4*/*arg4*) | SN152 | As SN152, but *vps3::HIS1*/*vps3::*  *LEU2 MTL***a**/α | This study |
| FDZF259 | *vps3*/*vps3* **a** (*leu2*/*leu2*) | FDZF162 | As SN152, but *vps3::HIS1*/*vps3::*  *ARG4 MTL***a**/Δ | This study |
| FDZF250 | *vps3*/*vps3* α (*arg4*/*arg4*) | FDZF158 | As SN152, but *vps3::HIS1*/*vps3::*  *LEU2 MTL*Δ/α | This study |
| FDZF512 | *vac1*/*vac1* **a**/α (*arg4*/*arg4*) | SN152 | As SN152, but *vac1::HIS1*/*vac1::*  *LEU2* *MTL***a**/α | This study |
| FDZF531 | *vac1*/*vac1* **a** (*leu2*/*leu2*) | SN152**a** | As SN152, but *vac1::HIS1*/*vac1::*  *ARG4 MTL***a**/Δ | This study |
| FDZF533 | *vac1*/*vac* α (*arg4*/*arg4*) | FDZF512 | As SN152, but *vac1::HIS1*/*vac1::*  *LEU2* *MTL*Δ/α | This study |
| FDZF518 | *pep12*/*pep12* **a**/α (*arg4*/*arg4*) | SN152 | As SN152, but *pep12::HIS1*/*pep12::*  *LEU2* *MTL***a**/α | This study |
| FDZF546 | *pep12*/*pep12* **a** (*leu2*/*leu2*) | SN152**a** | As SN152, but *pep12::HIS1*/*pep12::*  *ARG4 MTL***a**/Δ | This study |
| FDZF534 | *pep12*/*pep12* α (*arg4*/*arg4*) | FDZF518 | As SN152, but *pep12::HIS1*/*pep12::*  *LEU2* *MTL*Δ/α | This study |
| FDZF489 | *vps21*/*vps21*+*VPS21*p-*VPS21* **a** (*leu2*/*leu2*) | FDZF263 | As SN152, but *vps21::HIS1*/*vps21::*  *ARG4::VPS21p-VPS21-SAT1*  *MTL***a**/Δ | This study |
| FDZF475 | *vps21*/*vps21*+*VPS21*p-*VPS21*^S24N^ **a** (*leu2*/*leu2*) | FDZF263 | As SN152, but *vps21::HIS1*/*vps21::*  *ARG4::VPS21p-VPS21*^S24N^*-SAT1 MTL***a**/Δ | This study |
| FDZF473 | *vps21*/*vps21*+*VPS21*p-*VPS21*^Q69L^ **a** (*leu2*/*leu2*) | FDZF263 | As SN152, but *vps21::HIS1*/*vps21::*  *ARG4::VPS21p-VPS21*^Q69L^*-SAT1 MTL***a**/Δ | This study |
| FDZF491 | *vps9*/*vps9+VPS9*p-*VPS9* **a** (*leu2*/*leu2*) | FDZF213 | As SN152, but *vps9::HIS1*/*vps9:: ARG4::VPS9p-VPS9-SAT1 MTL***a**/Δ | This study |
| FDZF493 | *vps3*/*vps3+VPS3*p-*VPS3* **a** (*leu2*/*leu2*) | FDZF259 | As SN152, but *vps3::HIS1*/*vps3::*  *ARG4::VPS3p-VPS3-SAT1 MTL***a**/Δ | This study |
| FDZF548 | *vac1*/*vac1+VAC1*p-*VAC1* **a** (*leu2*/*leu2*) | FDZF531 | As SN152, but *vac1::HIS1*/*vac1::*  *ARG4::VAC1p-VAC1-SAT1 MTL***a**/Δ | This study |

Noble SM, French S, Kohn LA, Chen V, Johnson AD. 2010. Systematic screens of a *Candida albicans* homozygous deletion library decouple morphogenetic switching and pathogenicity. Nat Genet. 42**:**590-8.

Tao L, Zhang Y, Fan S, Nobile CJ, Guan G, Huang GH. 2017. Integration of the tricarboxylic acid (TCA) cycle with cAMP signaling and Sfl2 pathways in the regulation of CO_2_ sensing and hyphal development in *Candida albicans*. PLoS Genet. 13**:**e1006949.

| FDZF549 | *pep12*/*pep12+PEP12*p-*PEP12* **a** (*leu2*/*leu2*) | FDZF546 | As SN152, but *pep12::HIS1*/*pep12::*  *ARG4::PEP12p-PEP12-SAT1 MTL***a**/Δ | This study |
| --- | --- | --- | --- | --- |
