# Supplementary material for: The Vps21 signalling pathway regulates white-opaque switching and mating in Candida albicans
Source: Mycology. 2024 Jul 12;16(1):357–68. doi: 10.1080/21501203.2024.2376533 (PMC11899209; doi:10.1080/21501203.2024.2376533)
Supplement: Supplemental Material [file TMYC_A_2376533_SM4730.zip › Supplementary information.docx]

**Supplementary information**

**Figure S1. Relative transcriptional expression of the mating associated MAPK pathway genes in the WT control and mutant strains of the Vps21 signaling pathway.** Opaque cells of the WT control, *vps21/vps21*, *vps9*/*vps9,* *vps3*/*vps3*, *vac1*/*vac1*, and *pep12*/*pep12* mutant strains were incubated in liquid Lee’s glucose medium (pH 6.8) for 24 hours at 25 °C. Total RNA was extracted for quantitative RT-PCR assays. The expression level of *ACT1* was used for normalization. The average value of the WT control strain for each gene was set as “1”. “*” indicates significant difference between the WT control strain and mutant strain (*p* < 0.05, two-tailed Student’s *t*-test). Strains used: WT (FDZF171); *vps21*/*vps21* (FDZF266); *vps9*/*vps9* (FDZF212); *vps3*/*vps3* (FDZF250); *vac1*/*vac1* (FDZF533); and *pep12*/*pep12* (FDZF534). The mating type of all strains used was *MTL*Δ/α.

**Table S1. Strains used in this study**

**Table S2. Primers used in this study**
