# Supplementary material for: The Vps21 signalling pathway regulates white-opaque switching and mating in Candida albicans
Source: Mycology. 2024 Jul 12;16(1):357–68. doi: 10.1080/21501203.2024.2376533 (PMC11899209; doi:10.1080/21501203.2024.2376533)
Supplement: Supplemental Material [file TMYC_A_2376533_SM4730.zip › Table_S2_Primers_used_0507_2024.docx]

**Table S2. Primers used in this study**

| **No.** | **Name** | **Sequence (5’ to 3’)** | **Purpose** |
| --- | --- | --- | --- |
|  | *VPS21-*5’flank*-*F | ATCTTTTAGCATGACGATCC | *VPS21* deletion |
|  | *VPS21-*5’flank*-*R | CACGGCGCGCCTAGCAGCGGAATCCGAGAAACGATATCAC |  |
|  | *VPS21-*3’flank*-*F | GTCAGCGGCCGCATCCCTGCATGTGAATGGCCATGGTTAT |  |
|  | *VPS21-*3’flank*-*R | CAACTTTCAAGGTAGGCATT |  |
|  | *VPS21-*check*-*F | TGTTGAGCAAAGAATAGTGG |  |
|  | *VPS21-*check*-*R | AGTGAATCACCAGCTTATGG |  |
|  | *VPS21-*ORF*-*F | AGTATGAAATCTGGGACACT |  |
|  | *VPS21-*ORF*-*R | GGAGCATTAGCATTATCAGT |  |
|  | *VPS9-*5’flank*-*F | AATACTTTAGATGCCAAGGC | *VPS9* deletion |
|  | *VPS9-*5’flank*-*R | CACGGCGCGCCTAGCAGCGGAGAATGGGTGGAATTGATGT |  |
|  | *VPS9-*3’flank*-*F | GTCAGCGGCCGCATCCCTGCAATGGGTACGGATCCTTTAA |  |
|  | *VPS9-*3’flank*-*R | TGAAGATGACTTGGAATTGG |  |
|  | *VPS9-*check*-*F | AGATGATCGACTGAACATTG |  |
|  | *VPS9-*check*-*R | TTGTTACCTGGAGATGGAAT |  |
|  | *VPS9-*ORF*-*F | AATTCAGCCTTACATTCCAC |  |
|  | *VPS9-*ORF*-*R | TTGATCCATAGACGCAAATG |  |
|  | *VPS3-*5’flank*-*F | AGGATATCCAACAGGGTAAA |  |
|  | *VPS3-*5’flank*-*R | CACGGCGCGCCTAGCAGCGGACTATCCGAGATCAACGAAT | *VPS3* deletion |
|  | *VPS3-*3’flank*-*F | GTCAGCGGCCGCATCCCTGCAGCCGAAAATCTACGTACAA |  |
|  | *VPS3-*3’flank*-*R | GTTGCTGTGCCATTGTTTAT |  |
|  | *VPS3-*check*-*F | ATTGATTGCTGCCGATTTAG |  |
|  | *VPS3-*check*-*R | TACTATCCACAGGCAATGTT |  |
|  | *VPS3-*ORF*-*F | TTGGTCCAAGCTGTAATTAC |  |
|  | *VPS3-*ORF*-*R | ATAAAAGAAGCGCTACTGCA |  |
|  | *VAC1-*5’flank*-*F | AATTGGCCAAAATTCCTGAG | *VAC1* deletion |
|  | *VAC1-*5’flank*-*R | CACGGCGCGCCTAGCAGCGGATATCAATGTGGCCTGTAGT |  |
|  | *VAC1-*3’flank*-*F | GTCAGCGGCCGCATCCCTGCAGTGAATTAGGAGAGTTTGG |  |
|  | *VAC1-*3’flank*-*R | AATGAGTCGTCAAGTAAGCT |  |
|  | *VAC1-*check*-*F | TTGCATGTTGCTAATCTAGG |  |
|  | *VAC1-*check*-*R | AAAGTCGGGTCCTTTATGTT |  |
|  | *VAC1-*ORF*-*F | AGTGTCAAGAACGGTATAGT |  |
|  | *VAC1-*ORF*-*R | TTCATTGAACAACCTTTGCG |  |
|  | *PEP12-*5’flank*-*F | AGATTTTTGCTGACCAACTC | *PEP12* deletion |
|  | *PEP12-*5’flank*-*R | CACGGCGCGCCTAGCAGCGGATAAATGTGACCACAGGTAC |  |
|  | *PEP12-*3’flank*-*F | GTCAGCGGCCGCATCCCTGCATGTACAGAGGAGAAGTTTG |  |
|  | *PEP12-*3’flank*-*R | TCTGCTAGTGATGATTTGTC |  |
|  | *PEP12-*check*-*F | AAGAGCTTCGACAATTATGC |  |
|  | *PEP12-*check*-*R | TATCAACAAGACATTGCTGG |  |
|  | *PEP12-*ORF*-*F | TGTCTGAAAGTTTAAGTGGC |  |
|  | *PEP12-*ORF*-*R | ATTCACTATGGAIGGACAAGT |  |
|  | *VPS21* COMP 5' forward *Sal*I | AATCAAGTCGACTACCGTGGTATCTGTTATAC | Construction of the complemented strain *vps21/vps21+VPS21* |
|  | *VPS21* COMP 5' reverse *BamH*I | AATCAAGGATCCATAACCATGGCCATTCACA |  |
|  | *VPS21* COMP 3' forward *Xho*I | AATCAACTCGAGATGTGAATGGCCATGGTTAT |  |
|  | *VPS21* COMP 3' reverse *Sal*I | AATCAAAGATCTatGTCGACTTACCCTTTGCAATATCAGC |  |
|  | *VPS21*^Q69L^-Rev | GCAAGAGAAGCAAAACGCTCCAACCCAGCAGTGTCCCAGATTT | Construction of the *VPS21*^Q69L^ active  and *VPS21*^S24N^ inactive strains |
|  | *VPS21*^Q69L^-Fwd | AAATCTGGGACACTGCTGGGTTGGAGCGTTTTGCTTCTCTTGC |  |
|  | *VPS21*^S24N^-Rew | ACAAATCGCAACACCAACGAATTCTTTCCTACTGCAGCTTCTC |  |
|  | *VPS21*^S24N^-Fwd | GAGAAGCTGCAGTAGGAAAGAATTCGTTGGTGTTGCGATTTGT |  |
|  | *VPS9* COMP 5' forward *Hind*III | AATCAAAAGCTTTGAAGATGACTTGGAATTGG | Construction of the complemented strain *vps9/vps9+VPS9* |
|  | *VPS9* COMP 5' reverse *Kpn*I | AATCAAGGTACCTTAAAGGATCCGTACCCATT |  |
|  | *VPS9* COMP 3' forward *EcoR*V | AATCAAGATATCAATGGGTACGGATCCTTTAA |  |
|  | *VPS9* COMP 3' reverse *Hind*III | AATCAAAAGCTTTTGTTACCTGGAGATGGAAT |  |
|  | *VPS3* COMP 5' forward *Sal*I | AATCAAGTCGACTCGCAGATTCTGGTTATTAC | Construction of the complemented strain *vps3/vps3+VPS3* |
|  | *VPS3* COMP 5' reverse *BamH*I | AATCAAGGATCCATTGCTGGTGCATATGCTAT |  |
|  | *VPS3* COMP 3' forward *Xho*I | AATCAACTCGAGATAGCATATGCACCAGCAAT |  |
|  | *VPS3* COMP 3' reverse *Bgl*II+*Sal*I | AATCAAAGATCTatGTCGACAATTTGGATAACCAGGTTGC |  |
|  | *VAC1* COMP 5' forward *EcoR*V | AATCAAGATATCTTGCATGTTGCTAATCTAGG | Construction of the complemented strain *vac1/vac1+VAC1* |
|  | *VAC1* COMP 5' reverse *Pst*I | AATCAACTGCAGATAAAGTGCCCGAAGATGAA |  |
|  | *VAC1* COMP 3' forward *Kpn*I | AATCAAGGTACCTTCATCTTCGGGCACTTTAT |  |
|  | *VAC1* COMP 3' reverse *EcoR*V | AATCAAGATATCAATCCAAGTCATCAAACCCA |  |
|  | *PEP12* COMP 5' forward *Kpn*I | AATCAAAAGCTTTTTGAACAATACAATTTGGTAG | Construction of the complemented strain *pep12/pep12+PEP12* |
|  | *PEP12* COMP 5' reverse *Pst*I | AATCAACTGCAGTGAAATATTAGTGAGTGCCG |  |
|  | *PEP12* COMP 3' forward *Kpn*I | AATCAAGGTACCCGGCACTCACTAATATTTCA |  |
|  | *PEP12* COMP 3' reverse *Hind*III | AATCAAAAGCTTTGTGGCAGTATTGAATGATC |  |
|  | *caSAT1-Kpn*I-F | GGTACCGATTCATCCCATTCATTCCAT | Amplification of *caSAT1* |
|  | *caSAT1- Kpn*I -R | GGTACCCGCTCTAGAACTAGTGGATCTGAAG |  |
|  | *caSAT1-Pst*I-F | CTGCAGGATTCATCCCATTCATTCCAT |  |
|  | *caSAT1-Pst*I-R | CTGCAGCGCTCTAGAACTAGTGGATCTGAAG |  |
|  | RT-*STE2-*F | TACTGGTTGGTATGATGGATC | Real-time PCR of *STE2* |
|  | RT-*STE2-*R | AAGGCAACAACAATCAATCC |  |
|  | RT-*STE3-*F | TGTTGGTAAGTTGGATGCTG | Real-time PCR of *STE3* |
|  | RT-*STE3-*R | TGCATATCTTGATCCTGTCAC |  |
|  | RT-*FIG1-*F | AGAAGCTATGACTTGGACAGC | Real-time PCR of *FIG1* |
|  | RT-*FIG1-*R | AGTGGTTGTTGTTGGTGTTG |  |
|  | RT-*FUS1-*F | TAGCAAAAGCTCTCCAAATG | Real-time PCR of *FUS1* |
|  | RT-*FUS1-*R | TGCGATGTAGATGGTACTTTC |  |
|  | RT-*MFA1-*F | ATGGCTGCTCAACAACAATC | Real-time PCR of *MFA1* |
|  | RT-*MFA1-*R | AACAGAACAAGTGGAACAGC |  |
|  | RT-*MF*α*-*F | TGACAGTAACCAAGTTGTTG | Real-time PCR of *MF*α1 |
|  | RT-*MF*α*-*R | AGCACCAGAGGTAAGAGTAG |  |
|  | RT-*CST20-*F | AGATTCATATTCTCCTGGCAC | Real-time PCR of *CST20* |
|  | RT-*CST20-*R | ACTGTAGAAGTCGATGTTCC |  |
|  | RT-*STE11-*F | TGTCGGTAATAGTGCTTCCAG | Real-time PCR of *STE11* |
|  | RT-*STE11-*R | TCTCAAGGTCGATCTATGTGC |  |
|  | RT-*HST7-*F | AGATGCATCTCCAGTCAAAT | Real-time PCR of *HST7* |
|  | RT-*HST7-*R | TGACTCACAATTACACCTGA |  |
|  | RT-*CEK1-*F | TCGTCAAGTTTCATTCAACG | Real-time PCR of *CEK1* |
|  | RT-*CEK1-*R | TGTAGCTACGTATTCTGTCATG |  |
|  | RT-*CEK2-*F | ATACATGCCTCTGGATTTGC | Real-time PCR of *CEK2* |
|  | RT-*CEK2-*R | ACCTTTGTTGGTAGAGATCTG |  |
|  | RT-*CPH1-*F | AACATACAATGGTGATCCTAC | Real-time PCR of *CPH1* |
|  | RT-*CPH1-*R | AAATCTCGTTCCAAGGCATC |  |
